# Assessment of foot and ankle muscle strength using hand held dynamometry in patients with established rheumatoid arthritis

**DOI:** 10.1186/1757-1146-6-10

**Published:** 2013-03-22

**Authors:** Matthew Carroll, William Joyce, Angela Brenton-Rule, Nicola Dalbeth, Keith Rome

**Affiliations:** 1 AUT University, Health & Rehabilitation Research Institute, Auckland, New Zealand; 2 University of Auckland, Auckland, New Zealand

## Abstract

**Background:**

The foot and ankle are frequently affected in patients with rheumatoid arthritis (RA). One of the negative consequences of RA on the physical function of patients is a decrease in muscle strength. However, little is known about foot and muscle strength in this population. The aim of the study was to evaluate significant differences in foot and ankle muscle strength between patients with established RA against age and sex-matched controls using hand-held dynamometry.

**Methods:**

The maximal muscle strength of ankle plantarflexion, dorsiflexion, eversion and inversion was assessed in 14 patients with RA, mean (SD) disease duration of 22 (14.1) years, and 20 age and sex-matched control participants using hand-held dynamometry.

**Results:**

Significant differences were observed in muscle strength between the two groups in plantarflexion (*p* = 0.00), eversion (*p* = 0.04) and inversion (*p* = 0.01). No significant difference was found in dorsiflexion (*p* > 0.05). The patients with RA displayed a significantly lower plantarflexion-dorsiflexion ratio than the control participants (*p* = 0.03).

**Conclusions:**

The results from this study showed that the RA patients displayed a significant decrease in ankle dorsiflexion, eversion and inversion when compared to the non-RA control group suggesting that foot and ankle muscle strength may be affected by the pathological processes in RA. This study is a preliminary step for the measurement of muscle impairments within the RA population.

## Background

Rheumatoid arthritis (RA) is a chronic multifactorial inflammatory disease which can run a variable course. Patients require different levels of support at different stages of the disease as joint deformity and damage occur over variable periods of time [1]. At diagnosis, 16% of patients with RA may have foot joint involvement increasing to 90% as disease duration increases [2]. Joint involvement in RA often leads to deformities and muscle weakness, which dramatically affect RA management and outcomes [3].

Muscle strength in RA, involving the knee extensors and flexors has been reported in the literature [4,5]. However, little attention has been given to the strength of foot and ankle inversion, eversion, plantarflexion and dorsiflexion. Knowing the strength of these muscles may be useful to monitor the different stages of RA progression in a clinical setting, characterise the natural history of the disease, verify the efficacy of therapies, and document the relationship between impairments and disabilities. The aim of this study was to evaluate differences in foot and ankle muscle strength between patients with RA and age and sex-matched control participants using hand-held dynamometry. It was hypothesised that patients with RA would be significantly weaker in foot and ankle muscle groups compared with controls.

## Methods

### Participants

Fourteen adult patients with a history of documented RA were recruited from the AUT Podiatry clinic in Auckland, New Zealand. All patients were diagnosed with RA according to ACR diagnostic criteria [1]. Twenty age and sex-matched participants with no history of RA or other rheumatic diseases were recruited from the community as the control group. Participants were excluded if they: reported an acute flare at the time of assessment, were under the age of 18 years old, had a history of previous foot or ankle surgery, had a lower limb amputation, reported a history of neuropathy, lumbar radiculopathy, diabetes mellitus, inflammatory rheumatic disease other than RA or endocrine arthropathies. The AUT University ethics committee approved the study and participants provided written informed consent.

### Clinical characteristics

Age, ethnicity, sex, occupation, disease duration, RA positive, CCP positive, X-Ray erosion, current CRP, Health Assessment Questionnaire (HAQ)-DI [6] and current pharmacological management that included non-steroidal anti-inflammatory drugs (NSAIDs), methotrexate, other disease modifying anti-rheumatic drugs (DMARDs), prednisone and biologic therapies were recorded for each patient.

### Instrumentation

Isometric muscle strength was measured using a CITEC hand-held dynamometer (CIT Technics, Groingen, the Netherlands). The hand-held dynamometer (HHD) measures the peak force produced by a muscle as it contracts while pushing against an object. A recent systematic review of HHD for assessment of muscle strength in the clinical setting found the instrument to be a reliable and valid tool [7].

### Procedure

Isometric muscle strength was assessed using the ‘make test’, whereby the examiner held the HHD stationary while the participants actively exerted a maximal force [8,9]. For all muscle strength testing each participant was positioned in a sitting position (hips flexed and knees extended) with feet over the edge of an examination table. The HHD was positioned against the lateral border of the foot distal to the base of the 5th metatarsal head to measure eversion; to the medial border of the foot, near the base of the 1st metatarsal head to measure inversion; against the metatarsal heads on the plantar surface of the foot to measure plantarflexion, and on the dorsal aspect of the foot proximal to the metatarsal heads to measure dorsiflexion. Each participant performed submaximal test movements for familiarisation prior to testing. Testing of each muscle group required a contraction of 5-seconds. Three repetitions were obtained for each muscle group, with a minimum rest period of 10-seconds between each contraction. All testing was conducted by one examiner (WJ).

### Data analysis

Sex, ethnicity, clinical characteristics such as current pharmacological management are described as a number (percentage). All other demographic characteristics are described as the mean (SD). Normality of data distribution was assessed using the Shapiro-Wilk test and Levenes test. Independent *t*-tests were used to determine statistical significance between the muscle strength of the cases and the controls. A Kruskal-Wallis test was used to compare the differences between the strength ratios of the antagonistic muscle groups: inversion-to-eversion and plantarflexion-to-dorsiflexion. All data were analysed using Statistical Package for Social Sciences (SPSS, version 19) with the alpha level set at 0.05.

## Results

Participant characteristics are presented in Table 1. A total of 34 participants (14 patients with RA and 20 non-RA controls), all European females, were recruited into the study. The Shapiro-Wilk test indicated the data was normally distributed and the Levenes test indicated homogeneity of variances between all four strength test movements. Results are presented in Table 2. Independent *t*-tests demonstrated significant differences between cases and controls for plantarflexion strength (*p* = 0.00), eversion strength (*p* = 0.04) and inversion strength (*p* = 0.01). A significant decrease in plantarflexion/dorsiflexion ratio was found between the cases and controls (*p* = 0.03), but no significant differences in the strength ratio of inversion/eversion was found between cases and controls (Table 2).

**Table 1:** Participant demographics and clinical characteristics

	**Cases**		**Control**		***p*-value**
Age, mean (SD) years	66.8	(15.0)	67.9	(12.9)	0.83
Body mass index, mean (SD), kg/m^2^	26.3	(2.4)	25.4	(4.1)	0.06
HAQ-DI, mean (SD)	0.54	(0.4)	0.1	(0.2)	< 0.01
Disease duration, mean (SD) years	22.0	(14.1)			
Rheumatoid factor positive, n (%)	12	(86%)			
Anti-CCP positive, n (%)	5	(36%)			
X-ray erosions, n (%)	8	(57%)			
CRP, mg/L mean (SD)	4.9	(4.5)			
**Medications**					
DMARD use, n (%)	9	(64%)			
NSAID use, n (%)	5	(36%)			
Biologic use, n (%)	2	(14%)			
Corticosteroid use, n (%)	5	(36%)			

**Table 2:** Muscle strength testing measured with HHD

**Movement**	**Cases mean (SD)**		**Control mean (SD)**		**p-value**
Plantarflexion (N)	175.4	(58.8)	273.3	(61.5)	<0.01
Dorsiflexion (N)	123.0	(51.1)	156.4	(47.0)	0.06
Inversion (N)	91.9	(35.6)	127.5	(35.8)	0.01
Eversion (N)	92.1	(45.6)	121.7	(33.2)	0.04
Plantarflexion/Dorsiflexion Ratio	1.5	(0.3)	1.9	(0.5)	0.03
Inversion/Eversion Ratio	1.1	(0.3)	1.1	(0.2)	0.80

## Discussion

The aim of study was to evaluate foot and ankle muscle strength in patients with RA and age and sex-matched controls. It was hypothesised that the foot and ankle muscle strength would be reduced in those with RA. This study has shown a significant reduction in plantarflexion, eversion and inversion muscle strength in patients with RA. It is not clear from our design whether foot and ankle muscle reduction precedes development of RA or is a consequence of the disease.

The gastrocnemius-soleus muscle group are strong plantarflexors of the ankle, with the tibialis posterior muscle being the most powerful invertor of the rearfoot and a contributor to ankle plantarflexion [10,11]. While speculative, the reduction of the gastrocnemius-soleus muscle group strength may lead to altered gait strategy. Previous studies have suggested that the weakening of the calf muscles is mainly a result of pain-avoiding strategies through altered patterns of muscular activity in RA patients [12-14]. Although the current study did not undertake 3D kinematic or kinetic analysis of the lower limb and ankle, a recent study observed abnormal tibialis posterior EMG activity in a cohort of patients with RA, and ultrasound confirmed tibialis posterior tenosynovitis in the presence of suboptimal biomechanics and moderate levels of tendon disease [10].

The strength ratios between muscle groups in RA have been reported with the quadriceps/hamstring strength ratio being the most commonly investigated [15]. Again, only limited information is available about the relationship between plantarflexion/dorsiflexion and inversion/eversion strength ratios in RA. We found plantarflexion/dorsiflexion being stronger in the control group compared to RA cases. We also found that the inversion/eversion ratios were essentially the same for the RA cases and for the control group. RA has been reported to be associated with metabolic changes leading to a loss of muscle mass and strength [16]. However, muscle-specific force and muscle activation patterns have been found to be normal in RA patients with stable disease, even with significant muscle loss [15].

The study has a number of limitations. HHD is widely used and has been shown to be reliable for testing muscle groups in long term chronic conditions in adults and children but not in rheumatic diseases. A major limitation of HHD is the reliance on the tester to hold the device to accommodate the force being generated by the person being tested [17]. Future studies should evaluate both the intra and inter-tester reliability of HHD in rheumatic diseases. Previous RA studies have reported loss of muscle mass, decreased physical activity, and immunological factors may combine with alterations in skeletal muscle properties that could result in decreased muscle strength and functional limitation [18,19]. Therefore, the results from the current study on muscle strength alone need to be carefully interpreted. The results we found suggest that in a heterogeneous disease such as RA fluctuating symptoms, particularly joint pain and effusions may lead to measurement error. We can postulate that the difference in plantarflexion/dorsiflexion ratios may in fact be as a result of metatarsophalangeal joint synovitis or pain/tenderness which may inhibit the ability to generate force. As our study design was case–control, not longitudinal, it is not possible to know whether progression of the muscle strength decline is linear. Generalisation of these results can only be tentative, as the sample size was small and the patients participating in our study represent individuals with established RA and were all women. In the current study we used the x-ray erosion scores and the positivity of rheumatoid factor and anti-CCP to determine that the RA cohort was well established. A limitation of the study was not using a disease activity measure score such as the DAS28.

Further work should target the relationship between foot and ankle muscle activity and foot and ankle characteristics that include foot pain, foot sensation and intrinsic muscle strength of the foot since a significant number of patients with established RA suffer from foot and ankle problems [20]. Future work should also consider a standardised protocol when using HHD to measure foot and ankle muscle strength in a clinical setting.

## Conclusions

The study adds to the limited amount of published information on foot and ankle muscle strength in patients with established RA. Because of the consistent muscle weakness reported in the RA literature, we hypothesised significant differences would be found between cases and controls. The results tentatively indicate that RA patients with established disease have reduced muscle strength in foot and ankle plantarflexion, eversion and inversion.

## Competing interests

MC, WJ, ABR, ND and KR have no competing interests to declare.

## Authors’ contributions

KR designed the study. WJ collected the data. MC, KR and ND conducted the statistical analysis. MC drafted the manuscript with assistance from WJ, ABR, ND and KR. All authors approved the final manuscript.
